# Network rewiring, adaptive resistance and combating strategies in breast cancer

**DOI:** 10.20517/cdr.2019.60

**Published:** 2019-12-19

**Authors:** Constance Gaya Cremers, Lan K. Nguyen

**Affiliations:** ^1^Department of Biochemistry and Molecular Biology, School of Biomedical Sciences, Monash University, Clayton, Victoria 3800, Australia.; ^2^Biomedicine Discovery Institute, Monash University, Clayton, Victoria 3800, Australia.

**Keywords:** Network rewiring, adaptive resistance, PI3K signalling, MAPK signalling, breast cancer, mathematical modelling, systems analysis

## Abstract

Resistance to targeted anti-cancer drugs is a complex phenomenon and a major challenge in cancer treatment. It is becoming increasingly evident that a form of acquired drug resistance known as “adaptive resistance” is a common cause of treatment failure and patient relapse in many cancers. Unlike classical resistance mechanisms that are acquired via genomic alterations, adaptive resistance is instead driven by non-genomic changes involving rapid and dynamic rewiring of signalling and/or transcriptional networks following therapy, enabled by complex pathway crosstalk and feedback regulation. Such network rewiring allows tumour cells to adapt to the drug treatment, circumvent the initial drug challenge and continue to survive in the presence of the drug. Despite its great clinical importance, adaptive resistance remains largely under-studied and poorly defined. This review is focused on recent findings which provide new insights into the mechanisms underlying adaptive resistance in breast cancer, highlighting how breast tumour cells rewire intracellular signalling pathways to overcome the stress of initial targeted therapy. In particular, we investigate adaptive resistance to targeted inhibition of two major oncogenic signalling axes frequently dysregulated in breast cancer, the PI3K-AKT-mTOR and RAS-MAPK signalling pathways; and discuss potential combination treatment strategies that overcome such resistance. In addition, we highlight application of quantitative and computational modelling as a novel integrative and powerful approach to gain network-level understanding of network rewiring, and rationally identify and prioritise effective drug combinations.

## Adaptive resistance to anti-cancer monotherapy

Cancer is a complex network disease in which cells have acquired the ability to divide and grow uncontrollably, usually through genetic alterations in specific genes^[1]^. The discovery of molecular drivers of cancer and development of targeted drugs against these molecules have truly transformed the treatment of cancer. Imatinib (Gleevec), an inhibitor that blocks the BCR-Abelson murine leukemia viral oncogene homolog 1 (ABL1) tyrosine kinase activated in chronic myeloid leukemia (CML), is an outstanding example of an effective targeted therapy. Despite initial successes however, the overall progress of targeted therapy in the clinic has been hampered by the emergence of drug resistance, especially to those administered as single-agents, often referred to as monotherapy.

Drug resistance is a complex phenomenon and a major cause of cancer treatment failure, leading to patient relapse, disease progression and death^[2]^. Broadly, resistance to anti-cancer therapies can be divided into 2 categories: intrinsic or acquired. The former indicates the pre-existence of resistance-inducing factors in the tumour even before drug administration, and thus the treatment is ineffective from the start. In contrast, acquired resistance develops during the course of treatment, typically following an initial period when the treatment is effective. To date, multiple direct and indirect mechanisms underlying drug resistance have been identified, including - poor drug influx or excessive efflux, inherent cellular heterogeneity within the tumour, drug inactivation and alterations of the drug targets, which can act independently or in combination to limit drug efficacy^[3]^.

Among the mechanisms of acquired resistance, development of secondary mutations of the drug targets that compromise binding or inhibition of the drug to the target has been probably the most well studied. Notable examples include the emergence of T790M mutation in epidermal growth factor receptor (EGFR) leading to resistance to gefitinib in EGFR-mutant lung cancer^[4]^, T315 in ABL1 causing imatinib/dasatinib resistance in acute lymphocytic leukemia and CML^[5,6]^; and ERBB2/HER2 truncation leading to trastuzumab resistance in ERBB2-positive breast cancer^[7]^. In addition to these genetic mechanisms, it has become increasingly clear that tumour cells also rely on a non-genetic and highly adaptive mechanism involving dynamic rewiring of cell signalling networks to circumvent the initial drug blockade. A distinguishing and remarkable feature of drug-induced “network rewiring” and ensuing “adaptive resistance”, compared to classical resistance mechanisms, is that they can occur extremely quickly and have been commonly observed within hours or days following drug treatment in cell and animal tumour models^[8]^ as well as in cancer patients^[9]^.

Given the great relevance of network-mediated adaptive resistance, an increasing number of studies have been undertaken that have shed new light on the underlying mechanisms of drug-induced network rewiring and illuminated common themes behind the cause of adaptive resistance. Here, we review recent and notable experimental studies in this area with a special focus on this adaptive resistance phenomenon to kinase inhibitors targeting the phosphoinositide 3-kinases (PI3Ks)/AKT/mTOR and receptor tyrosine kinase (RTK)/rapidly accelerated fibrosarcoma (RAS)-MAPK signalling pathways in breast cancer (BC). We discuss potential combination treatment strategies where additional targeted drugs are combined with the initial agent to overcome adaptive resistance caused by treatment of the latter alone. Furthermore, as signalling networks are highly complex systems due to an abundance of feedback regulation, pathway crosstalk and intricate post-translational modifications, in-depth understanding of signalling network rewiring requires new integrative and quantitative approaches that extend beyond experimental work alone. The rapid development of adaptive resistance under typically short-time scales also begs for a new perspective to interrogate drug response dynamically rather than just obtaining a static snapshot. To this end, we will highlight the application of systems-based approaches combining computational modelling with lab based experiment to cope with these challenges and advance the discovery of effective combination therapies.

## PI3K-AKT-mTOR and RAS-MAPK signalling pathways in BC: key drivers of oncogenesis

BC is the most common cancer among women, which accounts for about a quarter of all diagnosed human tumours^[10]^. Although early diagnosis and enhanced therapies have greatly improved the overall survival time, BC is still a leading cause of cancer-related death worldwide^[10]^. While BC is a common term referring to tumours originating from the breast, it is an extremely heterogeneous disease with multiple subtypes that are distinct in molecular characteristics, level of aggressiveness and association with patient outcome^[11]^. Depending on the molecular data and measuring techniques used, the subtyping of BC may differ slightly, but it is usually classified into several major subtypes based primarily on the status of 3 major cell-surface receptors: luminal A [estrogen receptor (ER) and/or progestogen receptor (PR) positive, HER2 negative]; luminal B (ER+ and/or PR+, HER2+); HER2-amplified (ER-, PR-, HER2+), and triple-negative BC (Basal-like or TNBC, ER-, PR-, HER2-)^[12]^. Targeted therapies are available for luminal A/B and HER2+ BC, however due to the lack of all three receptors TNBC currently has no targeted treatment options.

**Figure 1:**
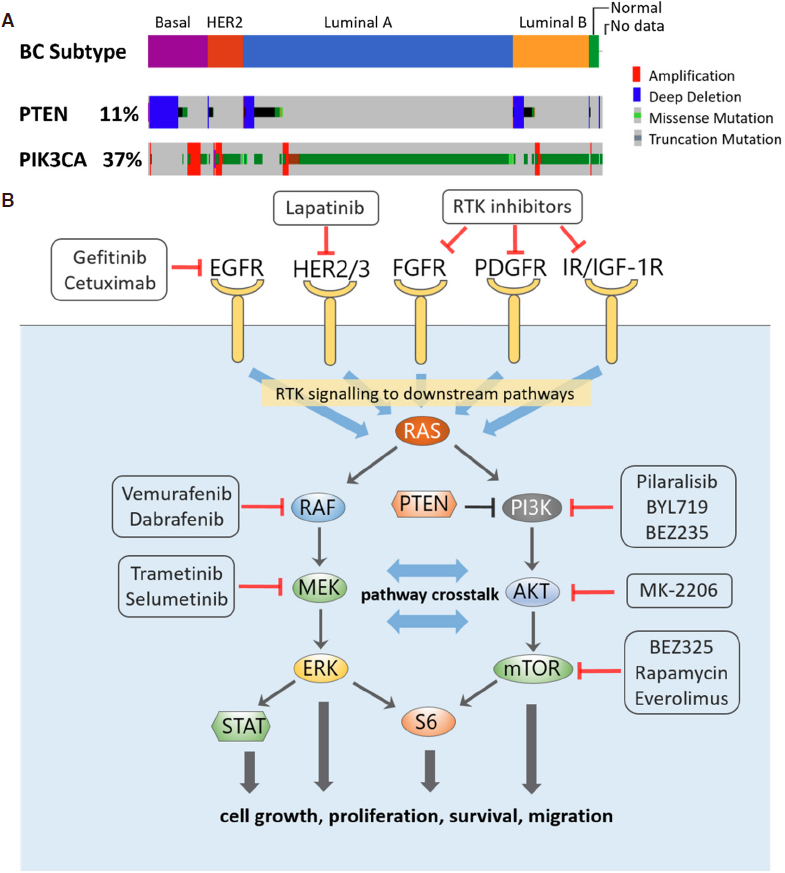
A: Frequency of alterations of PTEN and PIK3CA in breast cancer patients by subtype, analysed using the Pan-cancer Atlas dataset (*n* = 994) from The Cancer Genome Atlas Program (www.cbioportal.org); B: Signalling crosstalk between the PI3K and MAPK signalling pathways, with examples of targeted inhibitors directed at the network nodes

Advances in DNA sequencing over the past decade has enabled us to systematically study genetic alterations and their frequencies in cancer patients, leading to a better understanding of key cancer-driving signalling pathways. The PI3K-AKT-mTOR and RAS-MAPK signalling pathways are among the most frequently altered pathways across different cancer types including BC^[13]^. Located downstream of various RTKs, these are 2 major independent, yet highly interconnected, signalling cascades that critically regulate oncogenesis, reflected by their central roles in normal cell physiology^[14]^. The PI3K pathway is a prototypic survival pathway and is the most frequently dysregulated pathway in BC^[15]^, through a variety of genetic disruptions such as deletion of the tumour suppressor PTEN, oncogenic mutations in phosphatidylinositol-4,5-bisphosphate 3-kinase catalytic subunit alpha mutation (PIK3CA), and/or HER2 amplification^[16]^. Altered PI3K signalling, defined by alternation of one or more genes within the pathway, occurs across different BC subtypes, but most frequently in Luminal A, HER2+ and Basal-like BC (62%, 60% and 53%, respectively)^[13]^. The most common genetic alteration of this pathway are activating mutations in PIK3CA gene which encodes the p110α catalytic subunit of PI3K. Data compiled on the alteration frequencies using the CBioPortal Cancer Genomics shows that ~40% of BC patients had PIK3CA alterations, followed by 11% having PTEN deletion [Figure 1A]. Interestingly, while alterations in PIK3CA are primarily missense mutations and occur mostly in luminal A/B or HER2+ patients, loss of PTEN prominently happens in Basal-like/TNBC patients^[17]^.

Disruptive activation of the RAS-MAPK pathway, on the other hand, occurs in more than 30% of human cancers and is associated with increased risk of metastasis^[18]^. Activation of the pathway can be brought about by mutations in the core members RAS and RAF, but more often is due to alterations of upstream RTKs such as the ERBB family receptors, fibroblast growth factor receptor (FGFR) or MET. When this was taken into account, among the 10 most common oncogenic signalling pathways the RTK-RAS-MAPK pathway had the highest median frequency of alterations (46%) across all cancer types^[13]^. Of these, HER2+ BC has the third highest alteration rate in this pathway (82%), after only melanoma (94% altered) and the genomically-stable subtype of colorectal cancer (88%)^[13]^. EGFR, the most well-known upstream RTK of RAS-MAPK signalling, is frequently amplified in TNBC/basal-like subtype^[19,20]^, leading to pathway activation in ~50% of these patient groups^[21]^.

Due to their frequent alterations, targeting the receptor and protein kinase components of the PI3K and RAS-MAPK signalling pathways have been attractive therapeutic approaches for BC, reflected by an increasing array of targeted agents under active development and clinical testing^[22,23]^ [Figure 1B]. Yet, network rewiring and adaptive resistance represent major obstacles that limit the full clinical potential of these inhibitors. Below, we will first discuss these phenomenon observed for inhibitors targeting the RAS-MAPK pathway, followed by those targeting the PI3K pathway.

## Network rewiring in response to RAS-MAPK pathway inhibition: adaptive resistance mechanisms and proposed combination therapies

### MEK inhibition activates PI3K-AKT signalling by relieving negative feedback on ERBBs

As a central node within the RAS-MAPK signalling cascade, MEK represents a promising therapeutic target; however clinical studies of MEK inhibitors (MEKi) have shown only limited anti-tumour activity^[24,25]^. The earliest evidence of adaptive response to MEK inhibition in BC was reported almost a decade ago by independent groups^[26,27]^, where they found inhibition of MEK led to unexpected and rapid activation of PI3K-AKT signalling. To determine how this actually happened, Mirzoeva *et al*.^[27]^ performed a targeted reverse-phase protein array (RPPA) allowing for temporal response of ~30 pan-pathway signalling nodes to the MEK inhibitor U0126, using the TNBC MDA-MB-231 cells as a model system. Besides activated AKT which occurred as soon as 1 hour after drug treatment, RPPA revealed the inhibitor also induced marked activation of EGFR within the same time frame, which was even more pronounced in the presence of epidermal growth factor (EGF). MEK inhibition-induced AKT activation was confirmed in 5 (out of 8 tested) cell lines including TNBC and luminal lines, suggesting this is a common, yet cell-specific phenomenon. Since EGFR is an upstream input of PI3K signalling and ERK is a known negative regulator of EGFR^[28]^, the authors hypothesized that resistance to MEK inhibition is mediated by feedback activation of the PI3K pathway following relief of a negative feedback from MEK/ERK to PI3K/AKT via EGFR. Such feedback has been described previously^[29]^. In further support of this hypothesis, inhibition of EGFR effectively abolished the adverse AKT activation caused by MEK inhibition alone; and combined MEK-PI3K inhibition synergistically suppressed growth in 4 of the 11 BC cell lines tested^[27]^.

Similar findings were reported around the same time by Hoeflich *et al*.^[26]^, who provided additional *in vivo* evidence that dual MEK-PI3K inhibition was synergistic in reducing tumour growth in a MDA-MB-231 derived xenograft model of TNBC. A common conclusion reached by both studies was that basal-like/TNBC is particularly susceptible to MEK inhibition as compared to other BC subtypes, this however, seemed to be a weak association rather than a general rule as several TNBC cell lines, including MDA-MB-231, were among the most resistant cell lines against MEKi^[26,27]^. Lack of PTEN, which occurs in a subset of TNBC cell lines and promotes basal PI3K-AKT signalling, was attributed to enhanced resistance to MEKi-based therapy^[26]^. While MEKi-induced AKT activation tends to occur in BC cell lines having normal PTEN in these studies, it remains unclear if such adaptive response also happens in a PTEN-null background or if the already enhanced basal AKT activation would buffer the potential effect coming from breaking the MEK-EGFR-PI3K negative feedback.

The network rewiring induced by MEK inhibition that led to AKT activation is not exclusive to TNBC or HER2-negative BC. A few years later, a study from the Engelman group showed that this signalling remodelling also occurs in a range of HER2-driven cancers^[30]^, including breast and lung cancer. Importantly, this work provided critical mechanistic insights into the functioning of the MEK/ERK-to-PI3K feedback loop, which turned out to be mediated by ERRB3 (HER3), rather than EGFR directly. Specifically, MEK inhibition (by AZD6244/ selumetinib) activates AKT by inhibiting ERK activity, which blocks an ERK-mediated inhibitory threonine phosphorylation on the juxtamembrane domains of EGFR (T669) and HER2 (T677). Relief of this negative regulation by MEKi led to dramatic activation of HER3, enhanced binding of HER3 to GAB1 and PI3K, and AKT phosphorylation. Consistently, knockdown of HER3 abrogates this feedback and re-sensitises cancer cells to AZD6244 treatment. Although the previous studies did not examine HER3^[26,27]^, in hindsight the feedback activation of AKT seen in these works was also likely to be mediated by HER3, in addition to EGFR.

The above findings, collectively, may suggest that feedback activation of AKT is a common theme among breast and other cancers addicted to EGFR/HER2 and/or displaying over-activation of ERK signalling^[26,27,31]^, this however, is not the case. Indeed, when treating a panel of KRAS-mutant cell lines to MEK inhibitor, Turke *et al*.^[30]^ found that AKT was not adversely activated despite potent upregulation of phosphorylated ERBB3/HER3, indicating the MEK/ERK-ERBB3-PI3K feedback loop was not working under these conditions. This may be due to low levels of EGFR and HER2 in these cells, which were insufficient to transactivate ERBB3 to a level high enough for AKT activation. Another reason may be because the network circuitry is different and ERBB3 did not drive PI3K in these KRAS-mutant cell lines. In support of this, IGF-IR/IRS has been shown to be the major PI3K input in these cells^[32]^. While the exact cause(s) for the disconnect between ERBB3 and AKT activation requires further investigation, the above studies have demonstrated a highly dynamic and context-specific network rewiring mechanism to MEK inhibition involving the PI3K/AKT pathway, which underlies adaptive resistance to MEKi-based therapy.

### MEK inhibition drives extensive rewiring of the kinome and epigenomic networks

While inhibition of MEK had been known to acutely reprogram specific signalling networks, the extent and complexity of such reprogramming was only truly revealed in a seminal study in 2013^[8]^, thanks to advances in mass-spectrometry (MS)-based proteomics. Using a chemical proteomics approach that coupled kinase affinity capture with quantitative mass spectrometry, Duncan *et al*.^[8]^ was able to elucidate for the first time the kinome changes in response to MEK inhibition at a global level, in both cultured cells and genetically modified mouse models of TNBC. Remarkably, MEK inhibition by AZD6244 (and U0126) induced an extensive and dynamic remodelling of the cell signalling systems that extended far beyond ERBB/PI3K signalling, evident by large changes in expression and/or activation of > 140 kinases, from all major kinase subfamilies, within 24 h of treatment. These include a variety of pro-survival RTKs; PDGFRβ, VEGFR, AXL, HER2/3 and discoidin domain receptor family, member 1 (DDR1), and this inhibitor-induced RTK remodelling was accompanied by increased oncogenic signalling through the PI3K/AKT, JAK/STAT and MEK/ERK pathways, consistent with previous observations^[27]^. The results by Duncan *et al*.^[8]^ were significant as it revealed that selective perturbation of even a single node can trigger an extensive and rapid global response by the cancer cell signalling machinery, which counteracts the inhibitor’s effect.

**Figure 2:**
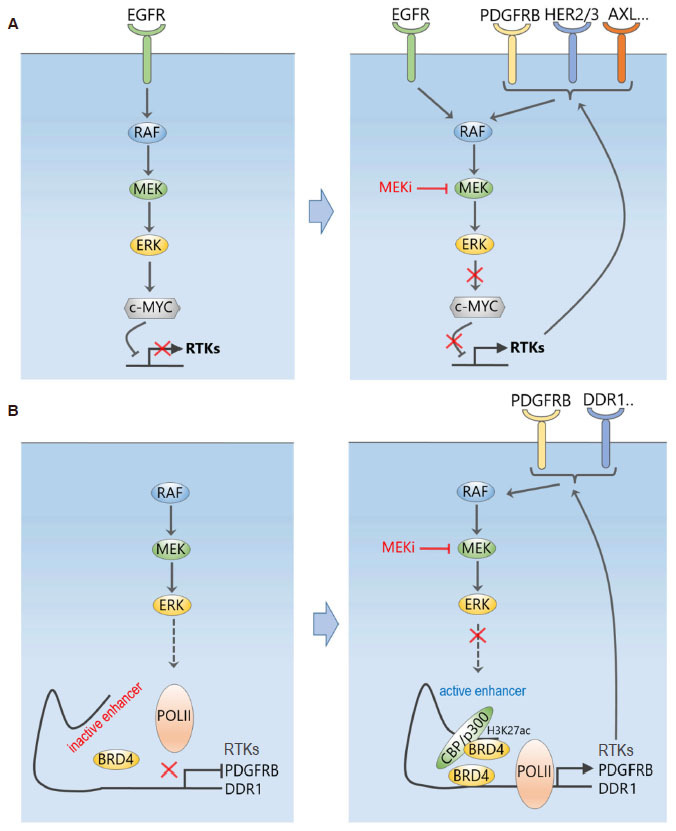
MEK inhibition dynamically reprograms the kinome and RTK signalling network. A: Inhibition of MEK disrupts a repressing transcriptional program exerted by the transcriptional factor c-Myc on the RTKs, which leads to induced expression and activation of an array of RTKs; B: MEK inhibition triggers a dynamic genome-wide enhancer formation with pronounced BRD4 density co-occupied with typical enhancer marks, causing increased expression and subsequent activation of RTKs, including PDGFRB, FGFR2, and DDR1

While defining the changes of signalling responses to targeted inhibitors is, nowadays, relatively straightforward with modern MS-based technologies like quantitative chemical proteomics, elucidating the underlying mechanisms of network rewiring is, however, more challenging. In addressing this, Duncan *et al*.^[8]^ found that the induced RTK expression/activation was due to disruption of a repressing transcriptional program exerted by the transcriptional factor c-Myc on the RTKs [Figure 2A]. As ERK phosphorylates c-Myc on S62 and enhances its stability, acute loss of ERK activity by MEKi treatment led to rapid c-Myc degradation and hence transcriptional de-repression of RTKs and their ligands that are negatively regulated by c-Myc. In support of this, RNAi-mediated knockdown of ERK or c-Myc induced similar RTKs as seen with MEKi, and blocking c-Myc degradation prevented the kinome reprogramming. Given c-Myc is not the only transcription factor regulating the induced RTKs, it is unlikely c-Myc degradation is the sole mechanism responsible for their transcriptional induction, yet this mechanistic insight offered valuable guidance for rational choice of combination therapy. For example, future selective inhibition of the E3 ligase(s) responsible for c-Myc degradation may help stabilize c-Myc and thus revert the MEKi-induced kinome remodelling. Until this is possible, the authors demonstrated proof of principle that combined treatment of MEK inhibitor selumetinib (AZD6244) with a pan-RTK inhibitor sorafenib synergistically reduced tumour growth in a mouse model of TNBC; albeit this combination is unlikely to be clinically useful due to the extensive off-target profile of sorafenib, which also targets RAF kinases.

To overcome this issue, a recent follow-up study^[9]^ has demonstrated that rather than trying to combat RTK upregulation using a secondary kinase inhibitor like sorafenib, the use of bromodomain and extra-terminal motif (BET) inhibitors (BETi), which targets bromodomain-containing proteins 2, 3 and 4 (BRD2/3/4), effectively and broadly prevented MEKi-induced transcriptional adaptation. This happened not only in TNBC cell lines, but also in patients following a small 7-day window-of-opportunity clinical trial of MEKi trametinib treatment, highlighting the significant clinical relevance of the findings. Mechanistically, the authors found MEKi induced an expansive, genome-wide and rapid remodelling of the epigenomic landscape^[9]^. BET family bromodomain proteins such as BRD4, bind to acetylated lysines of histone subunits or transcriptional factors to regulate transcriptional elongation through recruitment of positive transcription elongation factor (P-TEFb), an RNA polymerase II complex containing cyclin-dependent kinase 9 (CDK9) and Cyclin T1. Within 1-4 h of trametinib treatment, enhancers with pronounced BRD4 density co-occupied with typical enhancer marks were formed genome-wide, including at sites proximal to RTK loci such as *PDGFRB*, *FGFR2*, and *DDR1*, explaining their induced upregulation [Figure 2B]. Remarkably, BETi reduced the total number of MEKi-induced enhancers near baseline level; and BETi JQ1 combined with trametinib durably and synergistically inhibited tumour growth in both orthotropic and syngeneic mouse models of TNBC^[9]^. Consistent with the proposed model of RTK upregulation, small-molecule inhibition of P-TEFb constituent CDK9, or BRD4-associated factor p300 abrogated adaptive RTK induction.

Overall, the above studies together have unveiled extraordinary adaptive reprogramming of cancer cells to targeted MEK inhibition at both epigenomic and signalling levels, the former initially triggered the latter, which in turn likely fuelled further epigenomic changes in a positive-feedback manner. Although they have provided major insights in our understanding of inhibitor-induced acute adaptation, key questions remain to be answered. For example, given that the discussed work has utilised only a handful of TNBC cell models, are the observed rewiring mechanisms conserved across different TNBC cells, and if so do they occur to a similar extent? Clues to these questions came from^[9]^ where it found that TNBC cells of a basal-like subtype failed to remodel the BRD4 epigenome following MEK inhibition, while cells of the claudin-low subtype displayed comprehensive *de novo* enhancer formation, suggesting remodelling is likely cell type and context specific. Are the observed transcriptional and signalling rewiring and their mechanisms unique to MEKi? Or will different sets of RTKs be induced by inhibitors targeting other kinases, e.g., PI3K or mTOR? We believe in-depth answers to these questions will require more systematic efforts involving the use of large cell line panels and diverse drug agents, which will better illuminate the level and extent of tumour context-specific plasticity in response to targeted treatment.

### SHP2 drives adaptive resistance in KRAS-mutant and ERK-dependent tumours

**Figure 3:**
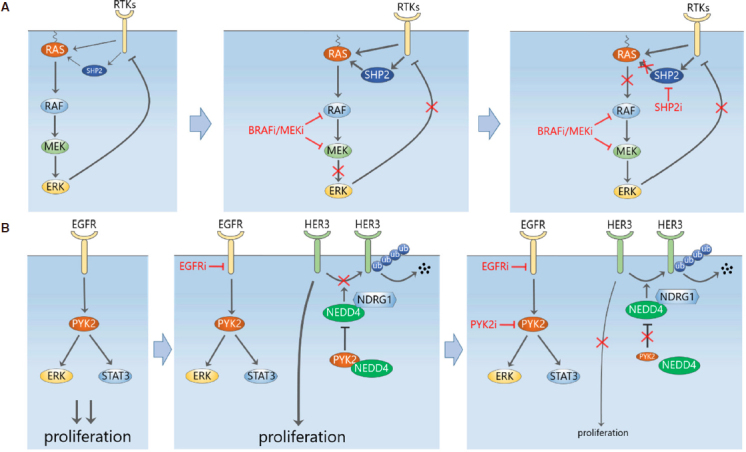
Adaptive upregulation of compensatory signalling limits the efficacy of EGFR-MAPK pathway inhibition. A: SHP2 is a convergent signalling node downstream of multiple RTKs. Inhibition of MEK induces SHP2 activation through increased RTK signalling and possibly other direct mechanisms, leading to ERK activity rebound. Inhibition of SHP2 prevents MEKi-induced ERK rebound through limiting the activity of RAS; B: upregulation of HER3 mediates adaptive resistance to EGFR in TNBC cells. PYK2 normally binds to the E3 ligase NEDD4 to inhibit it from degrading HER3. Inhibition of PYK2 destabilizes HER3 and resensitizes TBNC cells to EGFR inhibitors

In addition to overcoming MEK inhibitor resistance by targeting the induced RTKs directly with polypharmacology-based agents or preventing their transcriptional induction using BETi, inhibition of the convergent signalling “hubs” downstream of these RTKs also presents an attractive therapeutic strategy. This logic was successfully applied to Src homology region 2 (SH2)-containing protein tyrosine phosphatase 2 (Shp2), a phosphatase encoded by the gene *PTPN11*, which sits downstream of multiple RTKs and is critical for RAS activation. In 2018 and early 2019, five independent studies demonstrated that combining MEK inhibitor with a SHP2 inhibitor (SHP099) effectively abolished the adaptive resistance caused by single-agent MEKi treatment in a wide variety of RAS-mutant/amplified cancers, including pancreatic, lung and gastric cancer^[33-37]^. Biochemically, SHP2i prevents MEKi-induced ERK rebound through limiting the induced RAS-GTP loading mediated by the upstream RTKs^[37]^ [Figure 3A]. Interestingly, Fedele *et al*.^[37]^ further showed that this combined treatment also overcame adaptive resistance in RAS-normal TNBC cells. The effect on TNBC was subsequently solidified by Ahmed *et al*.^[36]^, who found the dual MEK-SHP2 inhibition profoundly inhibited both ERK signaling and cell growth in a panel of TNBC cell lines, including RAS-mutant and RTK-overexpressing TNBC cells, suggesting this combination provides a potential therapeutic strategy for TNBC patients. These results are in line with a previous finding that SHP2 promotes basal-like and TNBC^[38]^. Interestingly, not only MEK inhibition induced SHP2 activity, but also treatment of SHP2i alone was found to trigger a rebound of ERK^[37]^. This finding and evidence that SHP2 also acts upstream of RTKs (e.g., EGFR, MET and FGFR^[38]^) suggest the RTKs-SHP2-ERK circuitry is probably far more complex than currently known, and certainly more work is required for better mechanistic understanding.

While the collective evidence supporting MEK-SHP2 dual inhibition in KRAS-driven tumours is overwhelming, it is clear that this combination also works in additional tumour contexts, including those driven by ERK signalling either via BRAF mutations, overexpressed RTKs, or even under wild-type KRAS background^[33-37]^. The next key challenge in translating this combination therapy into the clinic will be identification of predictive biomarkers to guide patient selection for clinical trials. It appeared that patients having mutated RAS with high intrinsic GTPase activity (e.g., RAS G12C/S/A) are more sensitive to MEK-SHP2 co-targeting, while those with low GTPase-activity RAS mutants (e.g., Q61X) are more refractory to the regimen. Further, a high baseline level of phosphorylated SHP2 (e.g., pY542, indicative of SHP2 activity) seemed predictive of treatment sensitivity^[37]^; but as it remains unclear whether SHP2’s catalytic activity, its scaffolding function, or both are important for ERK rebound, the abundance of SHP2 may also serve as a good biomarker in certain contexts. Due to the complexity of the target network and highly context-dependent activity of MEK-SHP2 co-targeting strategy, determinants of its efficacy are probably multi-factorial and optimal companion biomarkers will likely involve multiple gene/protein indicators. We believe this issue is not unique to the MEKi+SHP2i combination, but rather will be a general rule for combination therapies.

### Extracellular RTK shedding contributes to adaptation to ERK signalling inhibition

Studies to date have primarily shown that tumour cells rewire their intracellular communication networks to adapt to drug challenge, extracellular mechanisms however, also contribute to such adaptive response. A variety of transmembrane receptors, including RTKs are known to undergo proteolysis via cleavage at extracellular sites mediated by metalloproteinases, such as A Disintegrin And Metalloproteinases 10 and 17 (ADAM10/17). Miller *et al*.^[39]^ found that MEK inhibition reduced the proteolytic shedding of multiple RTKs, including HER4, MET and most prominently AXL (an ADAM10/17 substrate) in melanoma and TNBC cells. Reduced RTK shedding increased the accumulation of full-length, signalling-competent RTKs on the tumour cell surface, which led to enhanced mitogenic signalling through downstream signalling such as the JNK/cJun pathway, thus evading the initial MEK inhibition. Consequently, combination of MEKi trametinib and AXL inhibitor R428 synergistically reduced tumour growth and metastasis in orthotopic TNBC (and melanoma) xenograft models derived from cell lines that showed increased surface AXL following MEKi. The findings by Miller *et al*.^[39]^ add an extra layer of complexity to the adaptation of cancer cells to targeted agents, and suggest that reduced RTK shedding may complement other bypassing mechanisms to reactivate oncogenic signalling. This is because many RTKs previously seen transcriptionally upregulated by MEKi, including PDGFRβ and VEGFR2, are also subject to shedding^[8]^. Figuring out which and how these different bypassing mechanisms co-operate under specific tumour contexts will be crucial in developing effective combination strategies to overcome them.

### TNBC circumvents EGFR inhibition through post-translational upregulation of ERBB3

It has been found that in TNBC patients EGFR inhibition is circumvented through HER3 upregulation^[40]^. Verma *et al*.^[41]^ recently showed that this HER3-mediated drug resistance was abolished by inhibition of the non-receptor tyrosine kinase proline-rich tyrosine kinase 2 (PYK2), thereby preventing the adaptive resistance to EGFR inhibition. They demonstrated that high expression of both PYK2 and EGFR is significantly associated with poor clinical outcome in TNBC patients, and combined targeting of EGFR and PYK2 was synergistic in blocking proliferation and inducing cell death of basal-like TNBC cells. Dual inhibition of EGFR and PYK2/FAK blocked key growth and survival pathways mediated by AKT, S6K, STAT3 and ERK1/2 activation. Importantly, the authors validated this drug combination *in vivo* by demonstrating it was able to attenuate tumour growth in a mouse xenograft model. These data suggest that EGFR-PYK2 co-inhibition provide a potential effective treatment for a subset of basal-like TNBC.

In addressing the mechanism underlying why PYK2 was a good EGFR co-target, the authors found that N-Myc Downstream Regulated 1 (NDRG1) enhanced the interaction of HER3 with the ubiquitin ligase neural precursor cell expressed developmentally down-regulated protein 4 (NEDD4), while PYK2, which interacts with NEDD4 and HER3, disrupted this NEDD4-HER3 binding. Inhibition of PYK2 thus facilitated the proteosomal degradation of HER3 and counteracted the increase in HER3 expression caused by EGFR antagonists [Figure 3B]. This provides a novel post-translational mechanism for drug-induced HER3 upregulation that is distinct from the previously discussed transcriptional induction of RTKs.

## Network rewiring in response to PI3K pathway inhibition: adaptive resistance mechanisms and proposed combination therapies

### Non-redundant functional roles of PI3K isoforms in normal and transformed cells

The PI3Ks generate lipid second messengers inside cells, which are essential for controlling cellular functions, including cell survival, proliferation, metabolism and migration. The complexity of PI3K signalling is, in part, due to existence of a large number (eight) of PI3K isoforms, grouped into three classes: class I, II and III, each generates different lipids - and controls different - biological aspects within the cell. While the reason(s) remain unclear, class I PI3Ks are the main PI3K genes found to be mutated in cancer, often at high frequency, and thus are the main PI3K isoforms currently pursued in anti-cancer drug development^[42]^. These PI3Ks are stimulated by tyrosine kinases, RAS and G protein-coupled receptors; and as such are often recruited by tyrosine kinase-based signalling networks, such as those activated by insulin and EGF. The class IA PI3Ks (PIK3Cα, PIK3Cβ, PIK3Cδ) exist as heterodimeric proteins made up of a regulatory p85 subunit (derived from three genes, *p85a, p85b* and *p55*) bound to one of three p110 catalytic subunits (p110α, p110β or p110δ, encoded by *PIK3CA*, *PIK3CB*, and *PIK3CD*, respectively).

The critical role of PI3K signalling in normal physiology and its frequent disruption in cancer has led to a major effort in developing inhibitors targeting the key kinase components of this pathway, in particular class I PI3Ks, AKT and mammalian target of rapamycin complex 1/2. To date, over 40 PI3K-signalling targeted inhibitors have been developed, which include isoform-selective PI3K inhibitors, pan-PI3K inhibitors, dual pan-PI3K and mTORC1/2 inhibitors, as well as specific inhibitors of mTORC1 and AKT. Although some of these agents such as the mTOR inhibitors (temsirolimus and everolimus) have already been approved for use in a number of cancers^[43,44]^, undue toxicities and emergence of resistance, including adaptive resistance to these inhibitors have significantly hampered their full clinical potential as single-agent therapies^[45]^. Clinical translation is further complicated by the poorly-understood observations that different p110 isozymes play non-redundant roles in cell transformation. For examples, while p110α is predominantly required for growth of tumours driven by RTKs, mutant RAS, and/or PIK3CA mutations, p110β is the dominant isoform in PTEN-deficient tumours^[46,47]^. Thus, compared to pan-PI3K inhibitors, isoform-selective PI3K inhibitors are likely less toxic to normal tissues. Although differing toxicities are associated with various classes of PI3K pathway inhibitors, common adverse events in BC include stomatitis, non-infectious pneumonitis, rash, hyperglycemia, and immunosuppression^[48]^. On the downside, the use of isoform-selective inhibitors may lead to compensatory upregulation of other PI3K isoforms that reactivate the pathway and limit the drug efficacy. Striking the right balance between efficacy and toxicity is a major challenge in translating PI3K inhibitors into the clinic.

### PI3K pathway inhibition reactivates AKT signalling through feedback upregulation of HER3 and other RTKs

In 2006, O’Reilly *et al*.^[49]^ provided one of the first pieces of evidence of a feedback bypass mechanism in response to PI3K signalling inhibition^[49]^. They showed that in BC cell lines with hyper-activated PI3K signalling, mTOR inhibition by rapamycin released the mTORC1-dependent suppression of insulin-like growth factor 1 receptor (IGF1R) and insulin receptor (IR), thus upregulating insulin receptor substrate 1 and restoring PI3K/AKT signalling^[49]^. This drug-induced relief of the mTORC1-to-IRS1 negative feedback largely explained the modest anti-tumour activity by rapamycin and mTOR inhibitor analogues seen in the clinic. A few years later, Chakrabarty *et al*.^[50]^ demonstrated that inhibition of PI3K by XL147 (pilaralisib), a highly selective pan-inhibitor of class 1A PI3Ks (α, β, γ, and δ), induced upregulation and activation of HER3 and other RTKs, including IR, IGF1R and FGFRs in HER2-overexpressing BC cell lines, which eventually reactivated PI3K/AKT signalling. The same changes were not due to off-target effects as they were also observed with another pan-PI3K inhibitor BKM120. The induction of these RTKs is explained in part by the relief of negative feedback from AKT to the RTKs via the forkhead box O (FOXO) family of transcription factors. Specifically, since AKT phosphorylates and inhibits FOXO via cytoplasmic sequestration^[51]^, AKT inhibition by XL147 released FOXO to the nucleus which was then able to transcribe the RTKs^[50]^. Importantly, because in HER2+ cell lines, HER2 is a major activator of HER3, the upregulation of HER3 expression resulted in significant HER2-mediated increase in its activity, ultimately triggering PI3K reactivation and limiting XL147’s efficacy. The authors went on to show that combinations of XL147 with HER2 antagonists (trastuzumab or lapatinib) were synergistic in delaying tumour growth in mice bearing xenografts derived from BT474, a HER2+/PIK3CA-mutant BC cell line. By utilising the same BC experimental models, very similar observations were also reported by Chandarlapaty *et al.*[52], but using AKT inhibitors instead of pan-class I PI3K inhibitors as in^[50]^. This similarity probably came from the fact that AKT is a common downstream node of the class I PI3Ks. While the above studies both suggested HER2 induced PI3K signalling via HER3, recent work showed HER2, when overexpressed, can directly activate PI3K/AKT signalling independent of HER3^[53]^. Regardless, these studies together highlight that combined PI3K/HER2 inhibition may be a potentially effective treatment for HER2-overexpresing BC patients.

Would PI3K/HER2 co-inhibition be useful even in non HER2-dependent tumours? There are several clues to this question. First, PI3K/AKT and FOXO-dependent upregulation of HER3 was found even following HER2 inhibition by lapatinib^[50,54]^. Remarkably, even dual blockade of HER2 with trastuzumab and lapatinib did not entirely eliminate the compensatory upregulation of HER3^[55]^. These studies suggest that - low levels of residual HER2 comparable to that in non-HER2-amplified tumours may be sufficient to phosphorylate and activate HER3, subsequently causing PI3K/AKT activation after PI3K/AKT or HER2 inhibition. Further, strong induction of common RTKs including IGF-1R, IR, HER3, Ephrin type-A receptor 7 (EphA7), and rearranged during transfection (RET) were seen following AKT inhibition in both HER2+ and non-HER2+ cell lines^[52]^. Collectively, these findings suggest that dual blockade of AKT and HER2 signalling may also be useful in non-HER2+ contexts. Indeed, combined AKT/HER2 inhibition was synergistic in suppressing tumour growth in mice bearing xenograft established from NCI-H292, a non-HER2 amplified lung tumour cell lines^[52]^. Provided toxicity is tolerable, dual combination of either PI3K or AKT inhibitors with HER3-neutralizing monoclonal antibody, or triple combination of PI3K/AKT, HER2 inhibitors and a HER3 antibody may be fruitful therapeutics for HER2+ as well as non-HER2+ cancers, as these combinations would more completely eliminate HER2-mediated HER3 activation. In support of this notion, combination of LJM716 (a HER3 neutralizing antibody) and BYL719 (a PI3Kα-specific inhibitor) inhibited AKT phosphorylation more potently than LJM716 or BYL719 alone and synergistically inhibited growth in a panel of HER2-overexpressing breast and gastric cancer cells^[56]^. Furthermore, in HER2-normal tumours where PI3K signalling is likely not driven by HER2 alone, depending on which upregulated RTKs, discussed above, are the primary input into PI3K/AKT signalling, co-inhibition of PI3K/AKT and such RTK(s) could also provide potentially effective therapies. Nonetheless such avenues clearly warrant further investigation in future research.

What about BC with co-alteration of HER2 and PI3K? Our analysis of data from TCGA (using Cbioportal) showed that almost one third of HER2-amplified BC patients also harbour PIK3CA mutation and/or amplification^[57]^. In another important study^[54]^, Chakrabarty *et al*.^[54]^ found that expression of H1047R PI3K (the most common PI3K mutation) in MCF10A human mammary epithelial cells, but not E545K PI3K, markedly upregulated the HER3/HER4 ligand heregulin (HRG). This provides, yet another mechanism where specific PI3K mutations further fuel the activation of HER3 mediated by HER2. As expected, the PI3K inhibitor BEZ235 markedly inhibited HRG and phospho-AKT (pAKT) levels and, in combination with lapatinib, completely inhibited growth of cells expressing H1047R PI3K^[54]^. These findings suggest that selection of drug combinations would need to take into account the specific mutation status of PIK3CA, as direct PI3K inhibitors may be required to inhibit the unwanted mutation-induced upregulation of ERBB ligands^[58]^. These results also point to the combined use of PI3K inhibitors and ERBB1-3-neutralizing antibody mixtures, such as pan-HER/ERBB which can simultaneously block targeted ERBB receptors and ligands^[59]^, as a potential therapy for BC tumours with HER2/PIK3CA co-alteration.

### PI3K pathway inhibition rewires ERK signalling through multiple mechanisms

While the above studies have primarily demonstrated that the PI3K/AKT pathway itself is a major escape mechanism to inhibitors targeting PI3K signalling, other studies also found that compensatory activation of ERK signalling provides another escape route. First, Carracedo *et al*.^[60]^ showed that inhibition of mTORC1 with rapamycin not only activated PI3K-AKT signalling, but also induced ERK phosphorylation in BC cell lines and tumour biopsies from patients treated with the drug. Rapamycin-induced ERK activation occurred in both normal and cancer cells lines, due to interference of a negative feedback from mTORC1/S6K to PI3K/RAS, most likely mediated via IRS1^[60]^. Later, Serra *et al.*^[61]^ demonstrated treatment of BEZ235, a dual PI3K/mTOR inhibitor, in HER2+ BC cells also led to potent ERK activation, but primarily through upregulation of the RTKs, particularly ERBB signalling. This mechanism of ERBB-induced ERK activity was confirmed as BEZ235 treatment combined with HER2/3 antagonists (lapatinib or trastuzumab) or MEK inhibitor (selumetinib) led to decreased ERK activity and improved anti-tumour activity *in vivo* compared to BEZ235 treatment alone^[60]^.

Does ERK activation depend on the inhibitor target? While BEZ235 was mainly used, Serra *et al*.^[61]^ also demonstrated ERK activation in response to a diverse range of agents such as pan-PI3K inhibitor (GDC-0941), p110α inhibitor (PIK-90), AKT inhibitor (MK-2206), as well as mTOR inhibitors (RAD001 and Torin1) in a couple of HER2+ BC cell lines including BT474, suggesting ERK activation is a broad consequence of PI3K signalling inhibition regardless of the targeted node^[61]^. This, however, is at odds with results from^[50]^, which reported no consistent ERK activation in the BT474 cell line in response to pan-PI3K inhibition. Because the data related to ERK activation in BT474 cells was discussed but “not shown” in^[60]^, we could not further analyse these findings. Additional clues to the above question came from Will *et al*.^[62]^ who showed that inhibition of PI3K, but not AKT, leads to the rapid, but transient inhibition of the RAS-ERK signalling axis in HER2+ BC cells; and this inhibition, though transient, is critical for the enhanced cell death caused by PI3K over AKT inhibitors. The authors posited that inhibiting PI3K causes the rapid inhibition of both AKT-mTOR and RAS-ERK signalling, whereas AKT inhibitors suppress only the former and, in fact, activate the latter. The discrepancies among the above studies deserve more investigation, which will offer more clarity on how dependent ERK activation is with regard to the specific inhibitors and/or the targets they inhibit.

In line with the above observations, a more recent study also reported induced ERK activation following prolonged HER2 inhibition with lapatinib in HER2+ BC cells, which was partially dependent on FOXO transcription factors^[63]^. Interestingly, the lapatinib-induced increase in ERK phosphorylation correlated with increased stability of c-Myc, suggesting that in this case, ERK activation was probably due to disruption of both the AKT/FOXO and ERK/c-Myc negative feedbacks to the ERBB receptor family caused by lapatinib-mediated acute AKT and ERK inhibition. Further, compensatory ERK activation was observed *in vivo* in a genetically modified mouse model of HER2+ breast tumour with co-existing PIK3CA (H1047R) mutation following inactivation of the oncogenic PI3K^[64]^. Collectively, the studies discussed here provide a strong rationale for targeting both the PI3K and ERK pathways in HER2+ BC. Activation status of these pathways, including whether or not the tumours harbour loss of PTEN and/or RTK overexpression, can influence therapeutic response and serve as useful biomarkers for therapy selection^[65]^.

### Network rewiring in response to PI3K isoform-specific inhibition

**Figure 4:**
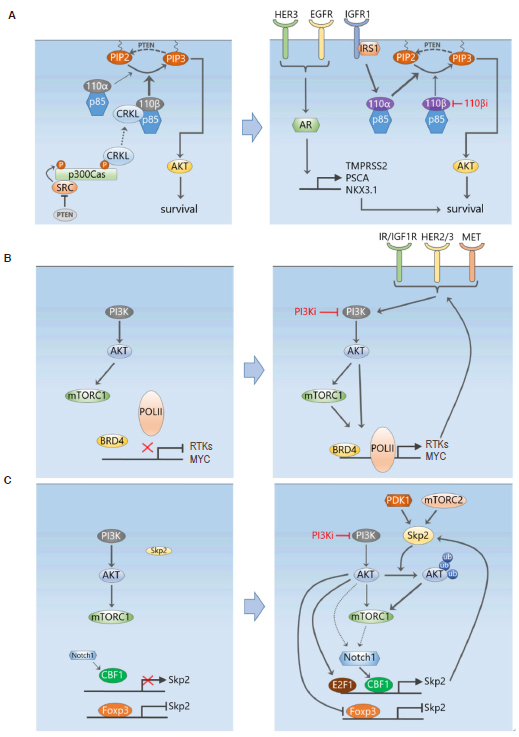
Selective adaptive resistance mechanisms in response to PI3K pathway inhibition. A: A PTEN/Src/p130Cas signalling axis activates CRKL/p110β in PTEN-deficient tumour cells, providing a link between PTEN loss and activation of p110β in these cells (left). Specific PI3Kβ inhibition cause feedback upregulation of IRS1 and IGF1R which then activates the PI3Kα isoform and results in a rebound of PI3K signalling following transient suppression (right). The androgen receptor downstream of several RTKs also provides another escape mechanism for continued survival following PI3K inhibition; B: similar to MEK inhibition, PI3K inhibition also reprograms the transcriptional machinery controlled by BRD4, leading to induced upregulation of multiple RTKs and MYC; C: a ubiquitin-based mechanism of adaptive resistance to PI3K inhibition mediated by the E3 ubiquitin ligase Skp2. PI3K inhibition leads to increased Skp2 expression and activity, which ubiquitinates and enhances the activation of AKT

The above studies have demonstrated adaptive resistance to pan-PI3K inhibitors, this however also occurred with more recently developed PI3K isoform-selective inhibitors. Schwartz *et al*.^[66]^ showed that PI3Kβ inhibition by AZD8186 only transiently suppressed PI3K signalling in PTEN-deficient breast (and prostate) cancer cells, with rapid rebound of PI3K/AKT signalling observed just 2 h after drug treatment. Interestingly, it was found that the rebound depended on activation of the PI3Kα isoform, which was caused by feedback upregulation of its activators IRS1 and IGF1R [Figure 4A]. Combination of AZD8186 with a PI3Kα isoform inhibitor (BYL719) or IGF1R/IR inhibitor (OSI-906) both significantly attenuated this AKT rebound and efficiently suppressed cancer cell growth^[66]^. In the same vein, the work by Costa *et al*.^[67]^ showed that PI3Kα inhibition by BYL719 initially abrogated PI3K signalling, but within 24 h induced a rebound in PI3K activation (indicated by the elevated phosphoinositide PIP3 level) in HER2+ or PIK3CA-mutant luminal BC cells^[67]^. Further analysis revealed that the elevated PIP3 was due to increased recruitment of the PI3Kβ isoform to HER3. As in^[66]^, co-inhibition of both PI3Kα and β significantly enhanced BC cell death and induced tumour regression *in vivo*[67]. These reciprocal feedback regulation among the PI3K isoforms highlight another intricate layer of the feedback circuitry controlling the PI3K signalling pathway. Systems-level understanding of complex feedback mechanisms and isoform-specific PI3K signalling will be important in identifying tumours susceptible to individual isoform inhibition, and informing appropriate combination therapy.

While it has been long known that in PTEN-deficient cancer cells PI3K signalling is driven by PI3Kβ, the mechanism linking PTEN loss with preferential p110β activation was only recently illuminated. Zhang *et al*.^[56]^ reported a role for the Crk-like adaptor protein (CRKL) in associating with and regulating p110β-dependent PI3K activity in PTEN-null cancer cells. Mechanistically, loss of PTEN activates Src, which in turn tyrosine phosphorylates the scaffolding protein p130Cas and phosphorylated p130Cas provides a platform for recruitment of CRKL that preferentially binds to p110β over p110α. Thus, a PTEN/Src/p130Cas axis activates CRKL/p110β in PTEN-null cancer cells [Figure 4A]. In support of this notion, the authors showed that Src inhibition co-operates with PI3K or p110β inhibition to suppress the growth of PTEN-null breast and prostate tumour cells^[56]^. However, further animal testing of these combinations are needed to confirm their synergistic effects in an *in vivo* setting.

To identify drugs that can be effectively combined with PI3Kβ inhibitor to comprehensively suppress PI3K signalling in PTEN-null tumours, Lynch *et al.*^[68]^ performed a cell proliferation based drug combination screen in a panel of PTEN-null TNBC, prostate, and renal cancer cell lines. Among the inhibitors targeting kinases known to be associated with resistance or feedback reactivation (e.g., mTOR, P13K, AKT, MEK and IGF-1R), the mTOR inhibitor vistusertib was found most effective when combined with the PI3Kβ inhibitor AZD8186 in suppressing cell proliferation. This combination also potently suppressed tumour growth *in vivo* in PTEN-null human tumour xenograft models^[68]^. Biochemically, combined mTOR/PI3Kβ inhibition efficiently inhibited PI3K/AKT signalling and cellular glucose uptake in the tested cell and animal models, which explained their superior efficacy. However, given the compensatory ERK activation is a known feature of PI3Ki-associated adaptive resistance, it was unclear if this happened in response to PI3Kβ inhibition and if the combined mTOR/PI3Kβ inhibition was also effective in eliminating it. More work is required to clarify this issue. Another point to note was that despite being deficient in PTEN, the TNBC cell line MDA-MB-468 failed to show similar synergistic anti-tumour benefit from the combined mTOR/PI3Kβ inhibition, the reason for which is unclear. This suggests that PTEN loss is not a sufficient biomarker *per se*, and more accurate biomarkers are required for PI3Kβ-based combination therapy.

As mentioned previously, unlike PTEN-null BC, in PIK3CA-mutant BC the p110α isoform predominantly drives PI3K signalling instead of p110β, leading to investigation of PI3Kα inhibitors such as BYL719 as potential therapeutics for these tumours. Elkabets and co-authors found that persistently active mTORC1 signalling was responsible for resistance to BYL719 despite efficient inhibition of AKT phosphorylation by the agent^[69]^. Inhibition of mTORC1 reduced resistance to PI3Kα inhibitors in *in vitro* and *in vivo*^[69]^. Shortly after, the same group further showed that PIK3CA-mutant cancer cells sensitive to BYL719 tend to potently inhibit phosphorylation of retinoblastoma protein (RB), a substrate of CDK4/6, whereas resistant cells failed to do so^[70]^. As expected, combined PI3K-CDK4/6 inhibition overcame BYL719 resistance, leading to tumour regressions in PIK3CA mutant xenografts. It is important to note, however, that both of these studies relied on resistant cell models established from prolonged exposure to BYL719, and so the observed resistance may involve epigenetic changes beyond adaptive network rewiring. Whether mTORC1 activation and/or RB phosphorylation take place dynamically in treatment-naïve PIK3CA-mutant cancer cells following p110α inhibition is unclear and requires further study.

### Overcoming adaptive kinome response to PI3K inhibition through BET inhibition

It has become clear from the above studies that similar to MEK inhibition, inhibition of the PI3K signalling pathway also triggers induction of a whole host of RTKs, many of which are also induced by MEKi. In light of the effectiveness of BET inhibition as a way to prevent RTK programming following MEKi, the use of BET inhibitors as part of combination therapies have been also explored in PI3K-driven tumours. Consistent with previous work, Stratikopoulos *et al*.^[71]^ showed that PI3K inhibition induces feedback activation of upstream RTKs and quick rebound of PI3K pathway activity. Importantly, they showed that BRD4 is key for these RTKs activation, with increased BRD4 occupancy observed at conserved regions upstream from the transcriptional start site of multiple RTKs and *MYC*, which was blocked by treatment with the BET inhibitor MS417 [Figure 4B]. Consequently, BET inhibitors inhibited the activation of AKT, mTOR, and MYC due to PI3K inhibition, and combined PI3K-BET inhibition sustained PI3K pathway inhibition and enhanced tumour cell killing in a variety of tumour models, including prostate cancer, melanoma and TNBC^[71]^. In another study, BET inhibition was also able to suppress lapatinib-induced transcriptional induction of a large portion of tyrosine kinases including those identified to contribute to growth (HER3, DDR1, FGFR2 and MET) in HER2+ BC cells^[72]^, preventing downstream SRC/FAK signalling and AKT reactivation.

Taken together, these findings suggest that combined kinase and epigenetic targeting can be a broader, more efficacious strategy to circumvent feedback-mediated resistance from inhibition of other kinases besides PI3K. This approach prevents adaptive resistance via kinome reprogramming by blocking transcription, generating the necessary sustained pathway inhibition, as well as overcoming the issue of heterogeneity in the adaptive kinome reprogramming response. Despite these promising results, further work will be required in additional models and in human clinical trials to determine the efficacy and safety of combining BET and PI3K inhibitors.

### Other adaptive resistance mechanisms to PI3K-AKT-mTOR signalling inhibition

Besides the PI3K and ERK pathways, other signalling pathways have been implicated in mediating network remodelling and adaptive resistance to PI3K signalling inhibition. Dual PI3K/mTOR inhibition using BE2235 was shown to induce IRS1-dependent activation of JAK2/STAT5 signalling, possibly via disruption of the mTORC1-IRS1 negative feedback^[73]^. In addition, BE2235 led to secretion of the pro-metastatic cytokine IL-8 that further activates JAK2/STAT5, driving resistance in TNBC. Accordingly, co-inhibition of PI3K/mTOR and JAK2 synergistically reduced cancer cell number and tumour growth, and also decreased tumour metastatic spread. In line with this finding, another study revealed that acquired resistance to PI3K inhibitors is mediated by feedback activation of IL6-STAT3 signalling, which triggered EMT and metastatic potential in human BC cells^[74]^.

In ER-positive BC, it has been recently shown that ER drives PI3K/AKT feedback activation induced by mTORC1 inhibition^[75]^. Inhibition of ER, IGF-1R/IR, or IRS-1/2 prevented the mTORC1 inhibition-induced AKT activation. This work suggests a strong rationale for combinations of anti-estrogens and mTORC1 inhibitors for ER-driven BC. Indeed, everolimus has been approved for treatment of recurrent/metastatic ER+ BC together with the aromatase inhibitor (AI) exemestane^[76]^.

While most of the adaptive resistance mechanisms discussed so far, are related to compensatory signalling activation mediated by phosphorylation, Clement *et al*.^[77]^ recently discovered a novel ubiquitin-based mechanism of adaptive resistance to PI3K inhibition. They found that in a subset of TNBC cell lines, PI3K inhibition, by BKM120 or PIK3CA depletion, ultimately promoted AKT reactivation in a manner partially dependent on the E3 ubiquitin ligase Skp2. Importantly, Skp2 expression robustly increased following PI3K inhibition, and levels of both Skp2 expression and AKT ubiquitination correlated with resistance to PI3K inhibitors. Depletion of Skp2 reduced AKT ubiquitination and activity, and inhibited the progression of BKM120-resistant BC xenografts^[77]^. Although the exact reason for PI3K inhibition-induced Skp2 expression is not yet clear, this could be due to inactivation of FOXO-mediated suppression or Skp2, and/or activation of Notch1, a known inducer of Skp2 [Figure 4C]. Given the complex feedback structure of this network, what is also unclear is the order of events leading to AKT reactivation following BKM120 treatment. Nevertheless, this study has unveiled a new PI3K-independent mechanism of adaptive resistance involving ubiquitin signalling. As ubiquitin is a major mediator of non-proteolytic cell signalling, we suspect this finding is only the tip of an iceberg of ubiquitin-related resistance mechanisms still to be discovered.

## Application of computational systems modelling to decipher drug-induced network rewiring and identify effective drug combinations

The experimental studies above (and others not discussed here due to space limitation), have revealed remarkable complexity into the mechanisms of targeted drug-induced network rewiring, which are highly diverse, dynamic and context-specific. This phenomenon reflects, in part, the presence of complex pathway crosstalk, intertwined positive and negative feedback loops, and post-translational modifications that together make signalling networks incredibly plastic and highly nonlinear. In-depth understanding of network remodelling therefore requires an ability to quantitatively describe drug-affected signalling-transcriptional networks and their dynamic behaviours overtime, which extends beyond experimental approaches alone. To this end, we believe systems-based approaches that integrate mathematical network modelling with experimental work will be essential for systematic interrogation of feedback and crosstalk disruption, dynamic drug response and ultimately drug-mediated network rewiring^[78-83]^. Mathematical models offer useful abstractions and powerful quantitative frameworks that enable us to validate our intuitive understanding, and gain new insights into these complex processes through formal analysis and predictive simulations^[84-87]^.

Moreover, mathematical modelling and model-based analysis can rationally inform suitable therapeutic targets and new drug combinations. While it is much more costly and practically challenging to screen vast number of possible target/drug combinations experimentally, predictive modelling, in principle, can be exploited to narrow down myriad possibilities and prioritise optimal combinations, thereby focusing experimental efforts only on these lead candidates^[88]^. We have recently demonstrated the validity of these concepts through model-based analysis of drug-induced signalling rebound in TNBC cells, and development of a computational drug combinations identication pipeline that enables *in silico* screening of numerous pair-wise drug combinations directed at signalling nodes and the ability to rank them by synergistic potential^[89]^. Applying this pipeline to a new mathematical model of EGFR signalling in TNBC led to predictions that combined inhibition of EGFR with PYK2, and to a lesser extent MET, displayed potent synergistic effects in suppressing oncogenic signalling. Experimental validation in TNBC cell lines and tumour xenograft confirmed these model predictions^[41,89]^. Further, unlike machine learning based approaches to drug combination discovery which often treat the target system as black-boxes^[90]^, dynamic modelling has the ability to offer mechanistic reasoning behind the synergistic effect of effective drug combinations, which are critical for assessing their application under different cellular contexts. Indeed, time-course simulations showed that EGFR-PYK2 co-inhibition was synergistic because it eliminated the adverse network rewiring and reactivation of STAT3 and ERK caused by either EGFR or PYK2 inhibition alone^[89]^.

The heterogeneity between cancer patients and their tumours leading to heterogeneous drug-induced network response poses a significant challenge for personalised cancer treatment. Here, mathematical modelling of biochemical networks further provides an effective approach to capture the patient-to-patient heterogeneity through incorporation of patient-specific - omics data and generation of patient-specific models^[91,92]^. These models can then be used to predict drug response^[91,93,94]^, design rational drug combinations^[92,95]^ and identify potential predictive biomarkers^[91,92]^ in a personalised manner. More interestingly, dynamic outputs from these computational network models can themselves serve as biomarkers^[96]^ that may be integrated with classical genes or protein-centric biomarkers for better personalisation of the treatment options. While mathematical modelling has been a highly useful tool for gaining systems-level understanding of signalling networks over the past decade, we believe future research priority should be placed on harnessing the translational capability of these models.

## Concluding remarks

This review has provided an integrative summary on the known mechanisms of adaptive resistance to inhibitors targeting the PI3K and RAS-MAPK pathways in BC (see Table 1 for a list of the major studies discussed). While these mechanisms appeared diverse in nature, several key themes have emerged. First, adaptive resistance occurs extremely quickly. Network-mediated activation of compensatory oncogenic signalling typically happens within hours of drug treatment in cancer cell lines. Although more work is required to monitor drug response *in vivo*, drug-induced network rewiring likely occurs in hours to days in animal models or patients, which is still much more rapid relative to the time typically needed for development of resistance due to genetic changes. This highlights the importance of the timing of drug combinations, which have been under-appreciated and under-studied so far. The fast timescale associated with adaptive resistance also implies the “wait-and-see” treatment strategies are not appropriate, and instead new treatments, such as combinatorial therapy, should predictively and pre-emptively prevent network adaptation before it takes place. Second, upregulation of RTKs is a recurring theme that applies to inhibitors targeting both pathways. Remarkably, common sets of RTKs tend to be induced by distinct inhibitors, indicating different inhibitors may utilise similar transcriptional machinery for RTK induction. Supporting this notion, combination of kinase inhibitors with epigenetic inhibitors such as those targeting BET have been shown to yield broad efficacy. It is likely that BET inhibitors may also be useful as part of combination treatments along with inhibitors for kinases other than those in the PI3K or ERK pathways. Third, adaptive resistance is primarily mediated by disruption of negative feedback loops. These feedbacks may have evolved to control important aspects of cell biology in non-transformed contexts^[97]^, but are hijacked by cancer cells to evade the drug effect. Moreover, although the studies reviewed here tend to focus on isolated feedback mechanisms, it is almost certain that they work together in any specific tumour setting, likely at differing intensities. Understanding which feedback (or combination of feedbacks) is dominant under which context(s) in mediating resistance will be critical in designing effective combination therapy to overcome it.

**Table 1:** Summary of selected network rewiring mechanisms in response to targeted inhibition discussed in this review

Targets	Drug agents	Rewiring mechanisms	Resistance-overcoming strategies	Ref.
MEK	U0126	Activated PI3K/AKT signalling, via MEK-EGFR-PI3K negative feedback	Combined MEK + PI3K inhibition	[26,27]
MEK	Selumetinib	Activated AKT signalling, via MEK/ERK-ERBB3-PI3K negative feedback	Combined MEK + ERBB3 inhibition	[30]
MEK	Trametinib	Upregulation/activation of multiple RTKs, via c-Myc degradation	Combined MEK + RTKs inhibition	[8]
MEK	Trametinib	Increased genome-wide BRD4-density enhancers leading to upregulation of multiple RTKs	Combined MEK + BET inhibition	[9]
MEK	MEK inhibitors	Activation of SHP2 signalling	Combined MEK + SHP2 inhibition	[33-37]
MEK	Trametinib	Reduced proteolytic shedding of multiple RTKs (AXL, HER4, MET), leading to incresed mitogenic signaling	Combined MEK + AXL inhibition	[39]
EGFR	Gefitinib	Enhanced HER3 signalling via PYK2	Combined EGFR + PYK2 inhibition	[41]
mTOR	Rapamycin	Activated IGF1R/IR via mTORC1-IRS1 negative feedback	Combined mTORC1 + IGF1R inhibition	[49]
PI3K/AKT	XL147 (Pilaralisib), BKM120 or AKT inhibitors	Upregulation and activation of RTKs (HER3, IR, IGF1R and FGFRs), partly via AKT-FOXO-RTKs negative feedback	Combined PI3K + specific RTK (e.g., HER3) inhibition	[50,52]
mTOR	Rapamycin	ERK activation via mTORC1-PI3K-Ras feedback	Combined mTORC1 and MAPK inhibition	[60]
PI3K/mTOR	BEZ235	ERK activation via ERBBs	Combined PI3K/mTOR and HER2/3 antagonists	[61]
PI3Kβ	AZD8186	IGF1R	Combined PI3Kβ + PI3Kα or PI3Kβ + IGF1R/IR inhibition	[66]
PI3K	PI3K inhibitors	Increased BRD4 occupancy at conserved regions upstream from the transcriptional start site of multiple RTKs and MYC	Combined PI3K + BET inhibition	[71]
PI3K/mTOR	BEZ235	IRS1-dependent activation of JAK2/STAT5 signalling	Combined PI3K/mTOR and JAK2	[73]
PI3K	BKM120	AKT reactivation via Skp2	Combined PI3K + Skp2 inhibition	[77]

In addition to sharing common features, specific mechanisms of adaptive resistance also display distinct properties depending on the targets and/or specific inhibitors used. For example, PI3K and AKT inhibition may trigger very different rewiring mechanisms by invoking different feedback loops. Importantly, many of the issues raised here can only be understood at the network level aided by mathematical and computational models of these networks. Thus, systems approaches that embrace predictive and quantitative modelling will be essential for future research into understanding network-mediated adaptive resistance and developing therapeutic strategies to combat adaptive resistance.

## Declarations

### Copyright

© The Author(s) 2019.
